# Testing of a treatment planning system with beam data from IAEA TECDOC 1540

**DOI:** 10.4103/0971-6203.79688

**Published:** 2011

**Authors:** B. J. Healy, R. L. Murry

**Affiliations:** Radiation Oncology Queensland, Toowoomba, Queensland, Australia

**Keywords:** Photon dose calculation algorithms, quality assurance, treatment planning system

## Abstract

Quality assurance of external-beam treatment-planning systems is recommended, and this can be partly achieved with predefined type tests. The beam data and test geometries of IAEA TECDOC 1540 have been used to test the analytical anisotropic algorithm (AAA) and pencil-beam convolution (PBC) algorithm of the Varian Eclipse treatment planning system. Beam models were created in Eclipse for 6 MV, 10 MV and 18 MV from the available beam data. Twelve test geometries were re-created in Eclipse, and the differences between Eclipse dose calculations and dose measurements were recorded. The AAA algorithm generally performed better than the PBC algorithm for the 12 tests, but both algorithms did not meet predefined tolerances for asymmetric wedge fields. An in-house monitor unit check program based on collimator and phantom scatter factors with tissue-phantom ratios was also tested and its calculations were found to agree with measurements to within 3.2% for on-axis points.

## Introduction

Quality control testing of a treatment planning system (TPS) is recommended as part of a radiotherapy quality assurance framework.[1] The International Atomic Energy Agency (IAEA) Technical Reports Series (TRS) 430[2] recommends periodic tests such as external beam revalidation and monitor unit/time constancy. Also, during the commissioning phase, TRS 430 recommends algorithm-specific tests to confirm the appropriate response of the TPS algorithms to typical clinical plans. The IAEA has also compiled a series of type tests for TPS’s in TECDOC 1540.[3] These type tests were taken from tests originally compiled by Venselaar and Welleweerd.[4] The tests include geometries for asymmetric fields, heterogeneities, oblique incidence and overshoot, which judge the performance of dose-calculation algorithms in clinical situations. It is reasonable that these type tests could be used during TPS commissioning and quality control testing.

In this report, those type tests from IAEA TECDOC 1540 were used to characterize the performance of algorithms in the Varian Medical Systems (USA) Eclipse TPS and provide baselines for periodic quality control testing. Specifically in this report, both the pencil-beam convolution (PBC) algorithm and the anisotropic analytical algorithm (AAA) were tested for megavoltage photon beams. A simple in-house monitor unit (MU) check program based on the ESTRO Booklet 3[5] formalism was also tested. It is noted that testing of intensity-modulated radiation therapy (IMRT) beams is beyond the scope of this report. AAPM Report 119[6] is a useful resource for IMRT commissioning.

## Materials and Methods

The TECDOC 1540 is accompanied by a beam data set, which is freely available online.[7] The data set includes beam profiles, diagonal profiles and output factors for three nominal energies (6, 10 and 18 MV) measured on an Elekta (Sweden) linear accelerator. Beam data for the internal motorized wedge and block tray is also included. This data was used in the Varian Eclipse (v8.1) treatment planning system to create beam models of an Elekta linear accelerator for the PBC and AAA algorithms. TECDOC 1540 also gives 12 test geometries with up to 15 specific coordinates at which doses have been measured and recorded. Once the beam models were generated, a 40×40×40-cm cubic test phantom of density 1.0 g/cm^3^ was created in Eclipse, and the 12 test geometries were applied to the phantom. The TPS dose was recorded at each of the measurement points. Also available with the beam data online were Excel spreadsheets in which the TPS doses can be compared to the measured doses for the 12 test cases. The spreadsheets calculate differences between TPS doses and measured doses according to one of three formulae given in TECDOC 1540. The formulae are as follows:

(1) $$\mathrm{Difference}=100\;\frac{\left(D_{cal}\;-\;D_{meas}\right)}{D_{meas}}$$

(2) $$\mathrm{Difference}=100\;\frac{\left(D_{cal}\;-\;D_{meas}\right)}{D_{meas,\;\mathrm{CAX}}}$$

(3) $$\mathrm{Difference}=100\;\frac{\left(D_{cal}\;-\;D_{meas}\right)}{D_{meas,\;\mathrm{open}}}$$

Equation 1 is used for central axis measurement points; equation 2, for off-axis in-field points; whilst equation 3 is used for doses outside the field edge or under blocks. Note that the IAEA TECDOC 1540 includes beam data for a Co-60 unit, but this was not utilized in this work.

Table 4 in TECDOC 1540 (page 28) gives recommended tolerances for TPS calculations, which can be summarized as 2% for central axis points, 3% for off-axis points and complex geometries, 4% for combinations of complex geometries and up to 5% for doses outside the field edge. All doses calculated in Eclipse at the designated measurement points were entered in the appropriate Excel spreadsheets, and those points at which the difference in TPS-calculated dose and measured dose was outside the tolerance defined previously were highlighted. The TPS calculation parameters were those used clinically in our department, namely, a 2.5-mm dose calculation grid, number of arc segments equal to 14, and the modified Batho correction for the PBC algorithm.

Venselaar and Welleweerd[4] suggested a parameter termed *confidence limit* to summarize the performance of the TPS algorithm for each test geometry. The parameter is given by:

(4) Confidence limit = average devation + 1.5 × SD

where the average deviation is calculated from the individual differences from equations 1-3, and SD is one standard deviation of the differences. This parameter was calculated for all tests.

The IAEA beam data was also used to test an in-house monitor unit (MU) check program written using VBA code in Microsoft Excel. As per TRS 430, in our department every patient-plan MU calculation is checked independently with the MU check program. This program uses the formalism of ESTRO Booklet No. 3[5] with collimator and phantom scatter factors and tissue-phantom ratios. These parameters were generated from the supplied beam data. The MU check program uses radiological depth to account for heterogeneity and off-axis ratios from the largest square field when calculating dose at off-axis points. The MU check program does not calculate dose outside the field edge or in the build-up region, so the comparison to the measured points was not as comprehensive as for the TPS.

## Results and Discussion

**Figure 1:**
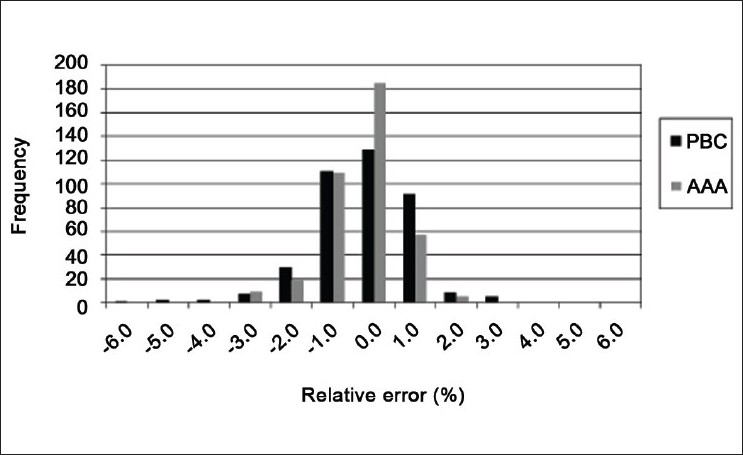
Histogram of difference between TPS-calculated dose and measured dose for the 10-MV beam energy. The data includes 384 individual measurement points but excludes results from test 12 (asymmetric wedge fields)

The TPS doses were calculated for the 12 test geometries, three photon energies (6, 10 and 18 MV) and two algorithms (PBC and AAA). The values of the confidence limit parameter are given in Table 1 for all tests. As expected, the superposition/convolution algorithm AAA generally performed better than the pencil-beam algorithm PBC in terms of the confidence limit parameter, but both algorithms performed poorly with the asymmetric wedge field (test 12). A histogram of the relative dose difference for 10 MV shows better agreement with the measurement of the AAA algorithm [Figure 1], with the results of test 12 excluded. Similar histograms were found for 6 MV and 18 MV (not shown). The AAA algorithm has previously been shown to perform better than the PBC algorithm, especially in the presence of heterogeneities.[8–10]

**Table 1:** Comparison of Eclipse-calculated dose and measured dose for twelve test geometries using the confidence limit parameter

*Test No. (as per TECDOC 1540)*	*Geometry*	*Recommended tolerance*	*Confidence limit value*					
			*6 MV*		*10 MV*		*18 MV*	
**			*PBC*	*AAA*	*PBC*	*AAA*	*PBC*	*AAA*
1	Square fields	3.0	1.9	1.5	1.8	1.5	1.9	1.8
2	Rectangular fields	3.0	2.5	1.6	2.4	1.6	3.2*	1.5
3	SSD = 85 cm	3.0	2.0	1.1	2.7	1.7	3.7*	1.0
4	Wedge field	4.0	3.2	3.9	1.9	1.7	3.3	2.1
5	Central block	4.0	3.4	1.0	4.6*	1.7	6.1*	3.2
6	Off-axis plane	4.0	2.1	1.7	1.9	1.3	1.5	1.3
7	Irregular field	4.0	2.5	1.2	3.6	1.9	4.4*	3.2
8a,b	Lung inhomogeneity	4.0	2.3	1.9	1.9	1.4	1.6	2.5
8c	Bone inhomogeneity	4.0	1.5	1.0	1.8	1.7	1.1	0.9
9	Oblique incidence	4.0	1.6	0.8	1.2	1.8	2.3	2.4
10	Overshoot	4.0	1.3	1.7	1.4	1.9	3.7	1.7
11	Asymmetric field	4.0	1.2	1.0	1.1	1.2	1.1	1.4
12	Asymmetric wedge field	5.0	10.9*	6.6*	10.7*	6.0*	9.5*	5.7*

PBC = Pencil-beam convolution, AAA = Anisotropic analytical algorithm

* Values of confidence limit outside tolerance

A characteristic of all the TPS test results was the worse performance of the algorithms in the build-up region when compared to the performance at depths beyond the depth of dose maximum. Each measurement set for the 12 geometries has points at 1-cm depth both on-axis and off-axis. Both algorithms often failed tolerance at 1-cm depth, but at no other depth. For example, for the 18-MV PBC beam model with a 10×10-cm field at 85 cm source-surface distance, the disagreement at 1-cm depth was up to 6.7%; whilst at greater depths up to 30 cm, the disagreement was, at worst, 1.2%. If the 1-cm depth data is excluded from analysis, the confidence limit parameter decreases from 3.7 to 1.2, which is well within tolerance. All the results outside tolerance for the 18-MV PBC beam model were due to excessive fails at 1-cm depth, apart from the asymmetric wedge field result. It can be argued that beam model inaccuracies in the build-up region are not of overall clinical significance, and that a distance-to-agreement tolerance rather than a local dose tolerance is more appropriate given the dose gradients in the build-up region.[4] Also, the difficulty of modeling electron contamination in the build-up region is likely to be a factor in the differences between TPS-calculated doses and measurements.[9]

A puzzling result was the poor performance of the 6-MV AAA beam model for the 9×9-cm wedge field (test 4), with a confidence limit of 3.9 compared to values of 1.7 and 2.1 for the 10-MV and 18-MV AAA models, respectively. Fails in tolerance were on the thick side of the wedge up to 4.8% at 35-cm depth. After a software upgrade, the same test was performed with the AAA algorithm version 8.9 rather than version 8.1. The confidence limit parameter was not improved, with a value of 4.8 and tolerance fails at depths 20, 25 and 35 cm. It must be pointed out that no attempt was made to alter default parameters in the wedge beam model, such as the relative intensity of the wedge scatter source, which could affect the final model. Also, the clinical significance of discrepancies at depths such as 35 cm is usually minor.

Whilst the AAA algorithm performed better than the PBC algorithm for the asymmetric wedge field (test 12), both algorithms failed the expected tolerance. A similar result was found previously for a range of TPS’s with confidence limits from 4.8 to 10.1.[4] A feature of the results was the fails when the asymmetry was towards the thick end of the wedge (parts b and e of the test) but passes in the higher dose regions in the central and thin parts of the wedge. This pattern is a consequence of the equation used to calculate the difference (equation 1), where the reference dose is taken as the local dose. This is in contrast to other suggestions for dose difference calculation[11] which rely on a reference dose at a normalization point (in this case, the depth of dose maximum). If the denominator in equation 1 is replaced by the measured dose at the depth of dose maximum in the equivalent symmetric wedge field, the number of measurement points outside 4% tolerance is reduced from 51 to 13 out of 105 points for the 6-MV PBC algorithm and from 21 to 3 out of 105 points for the AAA 6-MV algorithm.

The confidence limit parameter was calculated for the in-house MU check program, as shown in Table 2. The MU check program did not perform as well as the TPS in meeting tolerances. The MU check program did perform satisfactorily for measurement points on the central axis, with all check doses within 3.2% of the measured dose. Worse agreement was found for off-axis points, complicated by the fact that many off-axis points were close to the field edge. For example, in test 2.b, the off-axis point is 3 cm from the field edge; and in test 6, the off-axis plane is 1 cm from the field edge in the isocentric plane. The MU check program overestimates the dose at these points as it is expecting full scattering conditions. Finally it is noted that the MU check program performed better in terms of the confidence limit the higher the photon energy, i.e., best agreement was found for 18 MV. The reasons for this energy dependence are not clear.

**Table 2:** Comparison of MU check program calculated dose and measured dose for 12 test geometries using the confidence limit parameter

*Test No. (as per TECDOC 1540)*	Confidence limit value		
	*6 MV*	*10 MV*	*18 MV*
1	2.9	3.1	2.4
2	5.4*	3.0	2.7
3	4.6*	2.6	1.9
4	3.0	3.5	1.8
5	3.6	2.5	3.3
6	7.5*	6.4*	4.7*
7	5.1*	4.6*	2.5
8a,b	4.9*	4.1*	2.6
8c	2.6	2.8	1.7
9	3.9	2.2	0.9
10	6.4*	4.1*	4.8*
11	8.1*	7.0*	5.8*
12	16.4*	15.3*	14.6*

* Values of confidence limit outside tolerance

One limitation of the beam data set is its restriction to Elekta linear accelerators. Beam data for equipment such as the Varian Enhanced Dynamic Wedge, the Siemens Virtual Wedge, or Siemens double focus multi-leaf collimator would assist those departments who wish to test the strength of dose calculation algorithms for Varian and Siemens linear accelerators. Also, heterogeneities are only tested with simple cylindrical inserts in test 8. For more complex heterogeneous structures and complex beam arrangements, measurements in anthropomorphic phantoms are preferred, this being the subject of IAEA TECDOC 1583.[12] Finally, a couple of omissions were found with the downloaded beam data, namely, the file Gen_data.doc, which contains wedge factors and tray factors, was missing; and the file X10A404.HLX did not contain a profile for 10 MV, 40×40 cm, 20-cm depth, as expected.

Having established the beam models in Eclipse for the IAEA beam data set and having set baselines for performance against measurements, this work could be part of periodic quality control testing of the TPS. Also, algorithm upgrades as part of overall system upgrades could be tested against the baseline comparison data. Whilst there is no substitute for measurements on one’s own linear accelerators, the IAEA beam data set and point dose measurements would be useful for those departments that do not have access to phantom materials to test clinically relevant situations such as heterogeneity and overshoot on their TPS. Finally the IAEA material is obviously potentially useful as training material for physics registrars for training in beam modeling, plan generation and TPS quality assurance without interfering with departmental beam data and beam models.

## Conclusions

The PBC and AAA algorithms of the Varian Eclipse TPS have been tested using the beam data and test geometries of IAEA TECDOC 1540. The AAA algorithm met predefined tolerances in terms of agreement with measurement in test geometries, apart from asymmetric wedge fields. The PBC generally did not perform as well as AAA, complicated by fails against tolerance in the build-up region. An in-house MU check program performed tolerably for measurement points on the central axis but struggled with off-axis points. It is proposed that the test results could be used as baselines for routine quality control testing of the TPS.
